# Prevalence of Bladder and Bowel Dysfunction in Toilet-Trained Children With Urinary Tract Infection and/or Primary Vesicoureteral Reflux: A Systematic Review and Meta-Analysis

**DOI:** 10.3389/fped.2020.00084

**Published:** 2020-03-31

**Authors:** Jitendra Meena, Georgie Mathew, Pankaj Hari, Aditi Sinha, Arvind Bagga

**Affiliations:** Division of Nephrology, Department of Pediatrics, ICMR Center for Advanced Research in Nephrology, All India Institute of Medical Sciences, New Delhi, India

**Keywords:** dysfunctional elimination syndrome, vesicoureteral reflux, voiding dysfunction, constipation, urinary tract infection

## Abstract

**Introduction:** Urinary tract infection (UTI) in children leads to renal scarring in 10–15% of patients. Urinary tract anomalies and bladder and bowel dysfunction (BBD) are documented risk factors for recurrent UTIs. Estimates of baseline prevalence of BBD in children with UTI will help the clinician in the management strategy. Hence, a systematic review and meta-analysis was conducted to estimate the pooled prevalence of BBD.

**Methods:** MEDLINE, EMBASE, and CENTRAL (Cochrane Central Register of Controlled Trials) databases were searched for articles related to UTI, primary vesicoureteral reflux (VUR), and BBD. We included studies that provided prevalence of BBD in toilet-trained patients aged 1–18 years with UTI and/or VUR. BBD was defined based on clinical history or questionnaire or urodynamic studies. Two authors independently reviewed, assessed, and abstracted data from studies. Pooled prevalence was calculated based on a random effects model.

**Results:** Forty-three studies fulfilling the eligibility criteria were selected from a total of 1,731 studies. Among patients presenting with UTI without primary VUR, pooled prevalence of BBD was 41% (95% CI: 26–55; nine studies, 920 patients, *I*^2^ = 96.0%), whereas its prevalence in patients with primary VUR was 49% (43–56; 30 studies, 5,060 patients, *I*^2^ = 96.0%). Weighting by the study design and quality did not affect the prevalence. In patients with primary VUR, prevalence of BBD was higher in females (53%; 42–65) than in males (44%; 15–73). In studies where urodynamic study was used for the diagnosis of BBD, prevalence was 63%. The presence of BBD in patients with primary VUR increased risk of recurrent UTIs [relative risk (RR): 2.1; 1.7–2.5]. In five studies that reported separate data on constipation, pooled prevalence of constipation was 27% (16–37).

**Conclusion:** Almost half of the patients with primary VUR have BBD, and its presence increases the risk of recurrent UTIs. Trends of high BBD prevalence were also observed in patients presenting with UTI without VUR. These prevalence estimates suggest that all toilet-trained children presenting with UTI with or without VUR should be assessed for BBD, which will help in their further management.

## Introduction

### Rationale

Urinary tract infections (UTIs) are one of the most commonly encountered infections in childhood and may lead to long-term sequelae in a proportion of patients (1, 2). Whereas, presence of urinary tract anomalies is a known risk factor for recurrent UTIs in children, risk of recurrence is also influenced by age, gender, and bladder and bowel dysfunction (BBD) (3, 4). The term BBD is used to describe the spectrum of lower urinary tract symptoms accompanying bowel disturbance in the form of constipation and/or encopresis (4). BBD has also been reported as one of the important risk factors for recurrent UTIs in children. The risk of UTI is higher in patients with BBD and primary vesicoureteral reflux (VUR) than in patients with only VUR (5). Presence of BBD delays resolution of VUR and increases risk of UTI following reimplantation (6, 7). BBD has also been reported to impact the rate of breakthrough UTIs in patients with VUR who are on continuous antibiotic prophylaxis (5). As the presence of BBD in patients with UTI affects long-term outcomes, early recognition, and treatment are essential (6). In patients with UTI, variable prevalence (18–54%) of BBD has been reported in previous studies. Knowledge about the baseline prevalence of BBD in toilet-trained children presenting with UTI with or without primary VUR will help clinician in planning the management strategy for these patients (8). We performed a systematic review and meta-analysis to provide pooled estimates of prevalence of BBD in patients presenting with UTI and/or primary VUR.

### Objective

The aim of this study was to determine the prevalence of BBD in toilet-trained children with UTI with or without primary VUR.

### Research Question

What is the prevalence of BBD in toilet-trained children with UTI with or without primary VUR?

## Materials and Methods

### Study Design

A systematic review and meta-analysis was performed by review of observational and interventional trials published between January 1980 and December 2018.

### Participants, Interventions, and Comparators

All published data during 1980–2018 were searched for prevalence of BBD in toilet-trained children with UTIs with or without primary VUR. No interventions or comparators were assessed.

### Search Strategy

Protocol for the study was published (PROSPERO: CRD42019127086) and conducted in accordance with the Meta-analysis Of Observational Studies in Epidemiology guidelines (9). Two authors (JM and GM) independently performed literature search in MEDLINE, EMBASE, and CENTRAL (Cochrane Central Register of Controlled Trials) for original articles published, between January 1980 and December 2018. Search strategy design included patients aged 1–18 years with UTI and/or primary VUR. Search strategy was based on four basic groups of terminology: study population (pediatric/children/adolescent) and terms related to or describing the BBD, UTI, and VUR. Terminologies used for literature search were as follows: Bladder bowel dysfunction, dysfunctional elimination syndrome, dysfunctional voiding, lower urinary tract dysfunction, enuresis, urinary incontinence, urgency, overactive bladder, constipation, encopresis, fecal incontinence, vesicoureteral reflux, urinary tract infection, pyelonephritis, cystitis, pediatric, children, adolescent, prevalence, and incidence. Specific search strategies were created for each search engine by using MeSH term and terms described above (Supplementary Table 1). Electronic search was also supplemented by hand search of bibliographies of the included studies and relevant review articles.

### Data Sources, Study Selection, and Data Extraction

Predefined criteria were used for final selection of studies included in the review. All observational studies and controlled trials were included in this review if they (i) reported data on BBD prevalence in patients aged 1–18 years with UTI and/or primary VUR and (ii) defined BBD based on clinical history or questionnaire or urodynamic studies (UDSs). Conference abstracts were also included if they provided sufficient information on sample size, methods of data collection, case definition, and prevalence of BBD. Studies were excluded if they reported (i) BBD prevalence in 10 or less patients; (ii) patients with neurological abnormalities that affect normal functioning of bladder and bowel, or secondary VUR; (iii) non-toilet-trained children; and (iv) in languages other than English. A well-structured, standardized proforma was used for data extraction. Data included information for risk of bias assessment of the study, prevalence of BBD, author name, year of publication, journal, study setting and design, study population, baseline demographic characteristics, details of intervention, and control group (in case of randomized controlled trials), case definition of BBD, and recurrence of UTI. Any disagreement between two reviewers was resolved through discussion with the third author (PH).

### Statistical Analysis and Quality Assessment

The authors independently assessed the quality of articles using the Cochrane risk bias tool for randomized controlled trials. Quality of observation studies was assessed by using a risk of bias assessment tool developed by Hoy et al. for prevalence studies (10). We reviewed full-text articles to determine the following: (i) whether study participants are a close representation of true population; (ii) whether the method used for selection of the study participants was appropriate; (iii) whether data were directly collected from patients and their response rate; (iv) whether acceptable case definition and tool were used for defining BBD and UTI; and (v) whether appropriate numerator and denominator were used for calculating prevalence of BBD. Disagreement between two authors in assessment of risk of bias was resolved by the third author (PH).

Meta-analysis was performed using Stata version 14. We pooled data from individual studies using random effects model with assumption that BBD prevalence would be variable across the studies. Forest plots represent studies in order of year of publication. Heterogeneity in studies was explored by inspection of forest plot as well as using chi-square test on Cochran's *Q* statistics. Study heterogeneity was assessed by using the Higgins and Thompson *I*^2^ method (11). The *I*^2^ heterogeneity was categorized as follows: 0–50% low, 50–75% moderate, and >75% considerable heterogeneity. Sensitivity analyses were undertaken to investigate the individual study influence and the studies using only low risk of bias. A subgroup analysis was performed to explain heterogeneity and calculate prevalence of BBD by sex, study design (controlled trials and prospective and retrospective observation studies), and method of assessment.

## Results

### Search Results

**Figure 1:**
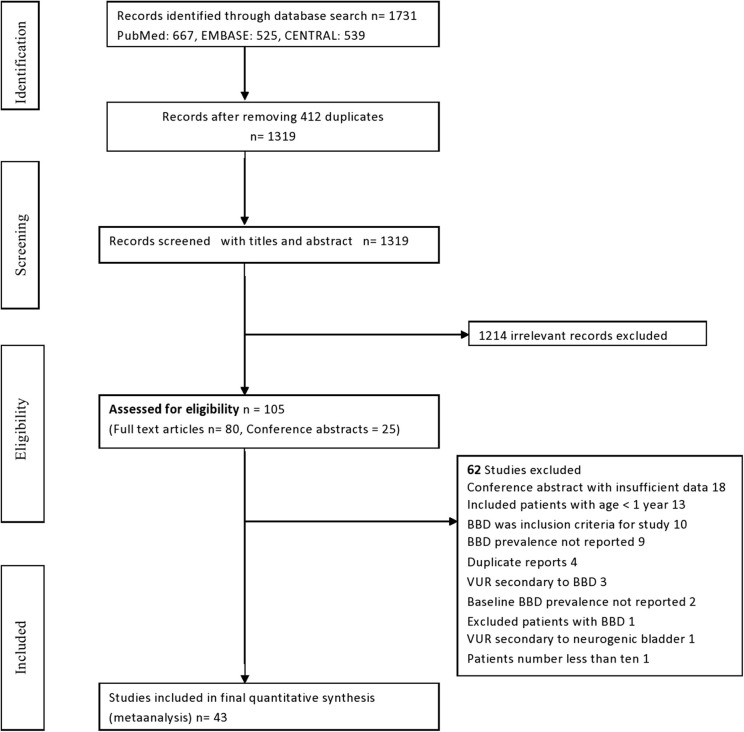
Systematic review flow diagram for selection of studies.

A total of 1,731 articles were identified through the search strategy in all databases (PubMed 667, EMBASE 525, and CENTRAL 539). There were 1,319 articles after removing 412 duplicates, and 105 of these articles were assessed as potentially relevant, for the systematic review, by screening through the title and abstract. Among these, 80 were full-text original articles, whereas the rest 25 were conference abstracts (Figure 1). We also screened the reference list of the full-text articles, but no additional article was identified through this process. Finally, 43 studies comprising 6,627 patients were selected for this review (Table 1).

**Table 1:** Basic characteristics of studies included in the systematic review.

**References**	**Country**	**Study design**	**Risk of bias assessment**	**Study group**	**Sample size**
Taylor et al. (12)	USA	Prospective	3	VUR	37
Seruca (13)	Portugal	Prospective and retrospective	3	VUR	43
Snodgrass et al. (14)	USA	Cross sectional	1	VUR	70
van Gool et al. (15)	USA	Prospective	1	VUR	310
Lipski et al. (16)	USA	Retrospective	2	VUR	30
David et al. (17)	France	Retrospective	3	UTI	30
Koff et al. (18)	USA	Prospective	1	VUR	143
Snodgrass et al. (19)	USA	Prospective	1	VUR	128
Trsinar et al. (20)	Slovenia	Prospective	2	VUR	94
Soygür et al. (21)	Turkey	Prospective	1	VUR	62
Willemsen et al. (22)	Netherlands	Prospective	1	VUR	102
Capozza et al. (23)	Italy	Prospective	1	VUR	180
Barroso et al. (24)	Brazil	Prospective	1	UTI	45
Mazzola et al. (25)	Switzerland	Retrospective	3	UTI	141
Vlajkovic et al. (20)	Yugoslavia	Prospective	1	VUR	74
Shaikh et al. (26)	USA	Prospective	1	UTI	123
Chen et al. (27)	USA	Retrospective	1	VUR	1721
Mingin et al. (28)	USA	Retrospective	2	UTI	12
Lavelle et al. (29)	USA	Prospective	2	VUR	52
Im et al. (30)	Korea	Retrospective	2	VUR	56
Colen et al. (31)	USA	Retrospective	2	UTI	132
Szymanik-Grzelak et al. (32)	Poland	Not clear	Unclear	VUR	150
Higham-Kessler et al. (33)	USA	Retrospective	2	VUR	80
Yucel et al. (34)	USA	Retrospective	2	VUR	92
Izquierdo and Luque Mialdea (35)	Spain	Prospective	2	VUR	63
Williams (36)	USA	Retrospective	Unclear	VUR	82
Sillén (37)	Sweden	Prospective	1	VUR	148
Whittam et al. (38)	USA	Retrospective	2		295
Altobelli et al. (39)	Italy	Retrospective	1	VUR	138
Hong et al. (40)	USA	Prospective	1	VUR	298
Palcic et al. (41)	Croatia	Retrospective	Unclear	VUR	92
Giuseppe et al. (42)	Italy	Retrospective	3	VUR	78
Öztürk et al. (43)	Not known	Not clear	Unclear	UTI	192
Akhavan et al. (44)	USA	Retrospective	2	VUR	78
Alexander et al. (45)	USA	Retrospective cohort	2	VUR	225
Heckler et al. (46)	USA	Prospective	2	VUR	169
Hoberman et al. (47)	USA	Randomized controlled trial	1	VUR	126
Cetin (48)	Turkey	Not clear	3	UTI	188
Chang et al. (49)	Taiwan	Retrospective	2	VUR	34
Chung et al. (50)	Korea	Retrospective	3	VUR	90
Arlen et al. (51)	USA	Retrospective	2	VUR	222
Shaikh et al. (5)	USA	Prospective	1	UTI	57
Sharif-Rad et al. (52)	Iran	Retrospective	1	VUR	225

*Risk of bias assessment (Low = 1, moderate = 2, High = 3)*.

### Study Selection and Characteristics

On the basis of patients enrolled, we categorized studies into two groups: (i) nine studies of patients with UTI and without primary VUR (5, 17, 24–26, 28, 31, 43, 53) and (ii) 30 studies of patients with primary VUR (12–16, 18–23, 27, 29, 30, 32–35, 37–42, 44–47, 49–52, 54). Three studies were included in the final systematic review as they reported data on rates of recurrence of UTI (15, 22, 45), and one study by Chung et al. provided data on constipation (50).

### Prevalence of Bladder and Bowel Dysfunction Among Patients With Urinary Tract Infection Without Primary Vesicoureteral Reflux

**Figure 2:**
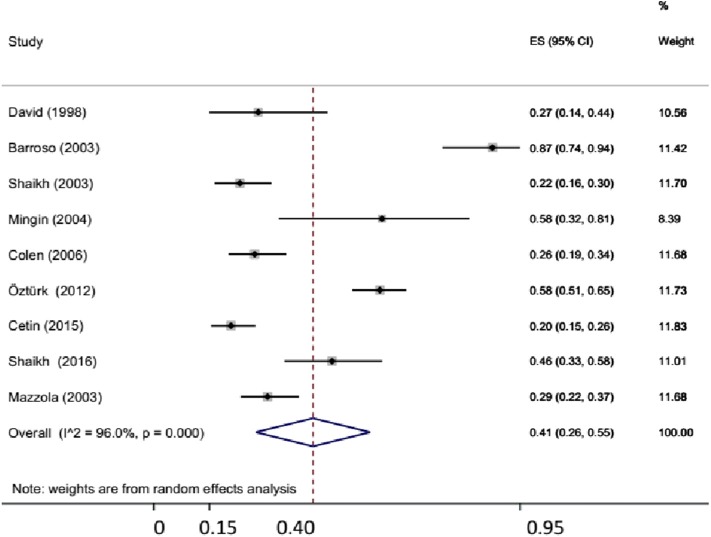
Prevalence of bladder dysfunction in children with urinary tract infection (UTI) without primary vesicoureteral reflux (VUR).

A total of nine studies comprising of 920 patients reported prevalence of BBD in toilet-trained children presenting with UTI without VUR (5, 17, 24–26, 28, 31, 43, 53). The pooled prevalence of BBD in patients with UTI was 41% (95% CI: 26–55%) (Figure 2). Owing to high heterogeneity (*I*^2^ = 95.99%), random effects estimates were used. Three studies reported separate data on prevalence of BBD in girls, at 41% (95% CI: 25–58%). None of the studies provided separate information on prevalence of BBD in boys. On a subgroup analysis, the prevalence of BBD was higher (51%, 10–93%) in prospective studies as compared with retrospective studies (35%, 21–49%).

### Prevalence of Bladder and Bowel Dysfunction Among Patients With Primary Vesicoureteral Reflux

**Figure 3:**
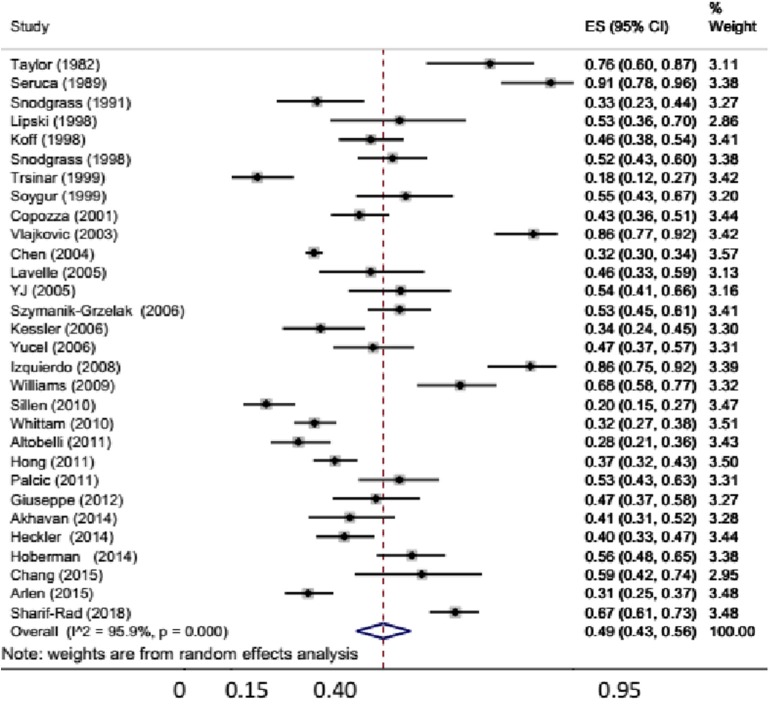
Prevalence of bladder dysfunction in children with primary vesicoureteral reflux (VUR).

Thirty studies comprising 5,060 patients reported prevalence of BBD in patients with primary VUR (14–16, 18–23, 27, 29, 30, 32–35, 37–42, 44–47, 49–52, 54–58). The pooled prevalence of BBD was 49% (95% CI: 43–56%) (Figure 3). In this group of studies, the heterogeneity was also high (*I*^2^ = 96%). Separate data for prevalence of BBD for boys were reported in five studies, with pooled prevalence of 44% (95% CI: 15–73%). Seven studies reported data for girls with BBD prevalence of 53% (95% CI: 42–65%). In studies with high risk of bias, the prevalence of BBD was high (71%), as compared with that in studies with low-to-moderate risk of bias (45% and 48%). No difference in BBD prevalence was observed when comparing prospective with retrospective studies. In eight studies that used UDS, the pooled prevalence of BBD was 63% (95% CI: 56–70%; *I*^2^ = 52.8%).

### Risk of Recurrence of Urinary Tract Infection in Patients With Bladder and Bowel Dysfunction

**Figure 4:**
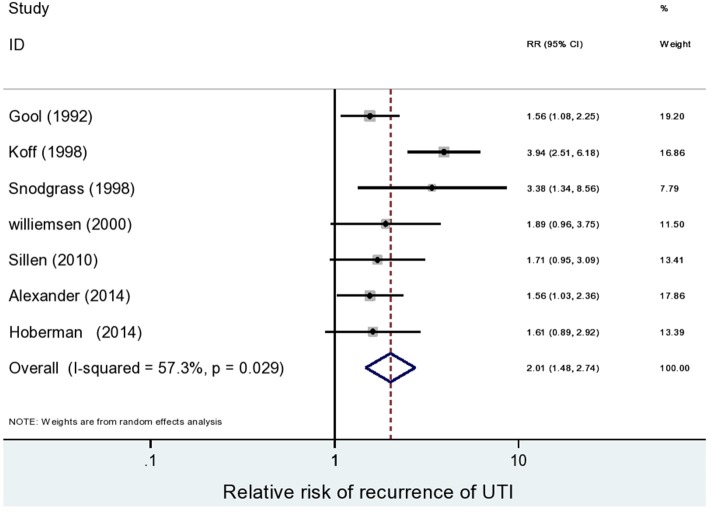
Risk of recurrence of urinary tract infection (UTI) in patients with vesicoureteral reflux (VUR) and bladder and bowel dysfunction (BBD) compared with patients without BBD.

Seven studies of patients with primary VUR explored the relationship between recurrence of UTI and BBD (15, 18, 19, 22, 37, 45, 47). A meta-analysis of these studies using random effects estimate showed that in the presence of BBD, the risk of recurrent UTIs was increased two-fold, as compared with that in patients without BBD (RR: 2.01; 95% CI: 1.47–2.74, *I*^2^ = 57.3%) (Figure 4). One study in patients with UTI without primary VUR reported that the presence of BBD did not significantly increase the risk of UTI (RR: 1.07, 95% CI: 0.51–2.23).

### Prevalence of Constipation

Two studies on patients with UTI showed that the pooled prevalence of constipation was 30% (95% CI: 25–36%) (24). Data for constipation were reported in 972 patients with primary VUR across seven studies (18, 21, 40, 47, 49, 50, 52). Pooled prevalence of constipation from these seven studies was 27% (95% CI: 16–37%).

### Risk of Bias

Quality of studies was assessed based on Cochrane risk bias tool for randomized trials, and a modified tool by Hoy et al. was used for observational studies (10). Based on these tools, seven studies were at high risk of bias, 17 at moderate risk of bias, and 15 at low risk of bias, whereas in four studies, risk of bias could not be assessed owing to insufficient information (Table 1).

## Discussion

### Summary of the Main Findings

Patients with bladder dysfunction are at increased risk of bowel dysfunction and vice versa. Anatomical and function interaction that leads to this increase risk has been well-established. BBD is an important risk factor for UTI in children, more so in the ones who are toilet trained. In a meta-analysis, the American Urological Association (AUA) guideline for the management of primary VUR showed that the presence of BBD significantly delayed resolution of VUR (7). In children presenting with UTI, who already possess a risk factor like primary VUR, the presence of BBD further increases risk of breakthrough UTI even while on antibiotic prophylaxis (5). Two recent studies support the notion that BBD predisposes patients for recurrence of UTI and increases risk of renal scarring as well (3, 5). Recently, a reanalysis of data from the RIVUR trial by Wang et al. showed that antibiotic prophylaxis is more beneficial in the group of patients with BBD compared with those without it (8). Hence, it is of paramount importance to assess children presenting with UTI for BBD even in the presence of other anatomical risk factors before deciding management strategy. In this review, we found that prevalence of BBD is slightly higher in patients with primary VUR than in patients with UTI without VUR. Within primary VUR, cohort girls had higher prevalence of BBD than boys. When BBD is assessed by more invasive tools like UDS, almost two-thirds of patients with primary VUR were detected to have BBD. In the present meta-analysis, we also found that the presence of BBD increases risk of recurrence of UTI by almost two times in patients with primary VUR. Functional constipation was documented in almost one-third of the patients with either VUR or UTI. Prevalence of BBD in patients with UTI without VUR is clearly higher, in the present meta-analysis, than in the general population of school-going children (20%) (55). This higher prevalence of BBD in children with UTI than the general population might point toward a strong association between BBD and UTI.

We found higher prevalence of BBD in patients with primary VUR than did a meta-analysis in the 2010 guideline for management of VUR by the AUA. In the meta-analysis by AUA, pooled prevalence of BBD in 15 studies was 31%. We used a predefined strategy for selection of the studies, which resulted in inclusion of different studies compared with those included in the meta-analysis by AUA. We also found that BBD is more common in girls with VUR, which could explain higher risk of breakthrough UTI in girls. Gaither et al. also reported higher risk of BBD in girls (56). Prevalence of BBD in patients with primary VUR, in the present meta-analysis, has varied from 18 to 91% (14–16, 18–23, 27, 29, 30, 32–35, 37–42, 44–47, 49–52, 54–58). This large variation in prevalence is likely due to multiple factors, which include characteristics of study population, study design, intervention, and assessment tool used for BBD. The largest randomized trial (RIVUR) in patients with primary VUR reported almost similar prevalence of BBD (56%) as in the present meta-analysis.

The relationship between VUR, BBD, and recurrent UTIs is complex and not so well-understood. A previous report from Shaikh et al. showed that patients with both VUR and BBD have the highest rate of recurrent UTIs than have patients with only VUR or BBD (5). This meta-analysis underscores the same fact and showed almost two-fold higher risk of recurrent UTIs in patients with coexisting VUR and BBD than in patients with VUR alone. Reanalysis of RIVUR trial showed that a subgroup of patients with both VUR and BBD had the most benefit from antibiotic prophylaxis. Significant reduction in recurrent UTIs following successful management of BBD with urotherapy has been reported, which again suggests a strong role of BBD in recurrence of UTI (57, 58). Hence, evaluation for BBD is essential while planning management for patients with primary VUR.

Prevalence of functional constipation was reported to be 9.5% in healthy children in a recent systematic review (59). In our review, prevalence of constipation was almost three times higher in children with or without VUR, suggesting that BBD is an important risk factor for UTI in toilet-trained children. A systematic review reported that 37–64% patients with functional constipation have lower urinary tract symptoms, hinting toward the association of BBD.

### Limitations and Strengths

There are few limitations of our systematic review. Large heterogeneity for final pooled prevalence could be considered a limitation; however, because there is no existing standardized diagnostic criterion to define BBD in children, we had to use all previous studies that provided data for BBD prevalence using various definitions. Second, the large heterogeneity could be because studies with all kinds of study design have been used in the present review, although in the sensitivity analysis, we could not find any major difference in various subgroups. Third, we had to exclude many studies that included infants, as these children aged <1 year are likely to be non-toilet trained and because diagnosing BBD in them is difficult. This exclusion criterion was defined *a priori*. Finally, we had limited our search to English-language databases only; hence, it might have resulted in exclusion of few studies published in non-English languages.

This systematic review has several strengths. First, we followed a rigorous methodology that included a comprehensive search of three major databases of medical literature, predefined protocol for study selection process, data extraction, and a statistical analysis that was registered in PROSPERO. We provided estimated pooled prevalence of BBD separately, in children with UTI only without VUR and other cohort of patients with primary VUR. We also showed that in patients with VUR, prevalence of BBD is higher in girls, which could explain higher number of recurrent UTIs in girls. Finally, we also assessed relative risk of recurrence of UTI in patients with both VUR and BBD and with VUR alone.

## Conclusions

In summary, this systematic review of currently available literature shows that BBD is common in toilet-trained children presenting with UTI with or without primary VUR. A subgroup meta-analysis also shows that functional constipation is common in these children, with almost every third child affected with it. We also found that the presence of both BBD and VUR doubles the risk of recurrence of UTI; hence, all children presenting with UTI should be carefully evaluated for presence of BBD and managed accordingly. As BBD is an important risk factor for UTI recurrence, in future, intervention trials for patients with primary VUR should be stratified as per presence of BBD.

## Data Availability Statement

All datasets generated for this study are included in the article/Supplementary Materials.

## Author Contributions

JM conceived of the presented idea, formulated the protocol, and wrote the manuscript. JM and GM did independent data collection and analysis. PH decided on conflicting data interpretation. AS, AB, and PH provided critical feedback. All authors discussed the result and helped shape the final manuscript.

### Conflict of Interest

The authors declare that the research was conducted in the absence of any commercial or financial relationships that could be construed as a potential conflict of interest.
