# Supplementary material for: Prevalence of Bladder and Bowel Dysfunction in Toilet-Trained Children With Urinary Tract Infection and/or Primary Vesicoureteral Reflux: A Systematic Review and Meta-Analysis
Source: Front Pediatr. 2020 Mar 31;8:84. doi: 10.3389/fped.2020.00084 (PMC7145391; doi:10.3389/fped.2020.00084)
Supplement: Supplementary file 1 [file Table_1.DOCX]

Supplementary table 1 Search strategy

| Pubmed | Search (((((((("Urinary Incontinence"[Mesh]) OR "Lower Urinary Tract Symptoms"[Mesh]) OR "Nocturnal Enuresis/epidemiology"[Mesh]) OR "Enuresis"[Mesh]) OR ((((((enuresis[Title/Abstract]) OR urgency) OR bed wetting) OR frequency) OR urinary incontinence) OR overactive bladder)) OR (((("Fecal Incontinence/epidemiology"[Mesh]) OR "Encopresis/epidemiology"[Mesh]) OR (( "Constipation/epidemiology"[Mesh] OR "Constipation/urine"[Mesh] ))) OR (((((((((bowel bladder dysfunction[Title/Abstract]) OR voiding dysfunction[Title/Abstract]) OR dysfunctional voiding[Title/Abstract]) OR dysfunctional elimination syndrome[Title/Abstract]) OR constipation[Title/Abstract]) OR fecal incontinence[Title/Abstract]) OR encopresis[Title/Abstract]) OR lower urinary tract symptoms[Title/Abstract]) OR lower urinary tract dysfunction[Title/Abstract])))) AND ((((((("Adolescent"[Mesh]) OR "Child"[Mesh]) OR (( "Pediatrics/diagnosis"[Mesh] OR "Pediatrics/diagnostic imaging"[Mesh] OR "Pediatrics/epidemiology"[Mesh] OR "Pediatrics/etiology"[Mesh] OR "Pediatrics/pathology"[Mesh] OR "Pediatrics/physiology"[Mesh] OR "Pediatrics/prevention and control"[Mesh] OR "Pediatrics/therapy"[Mesh] OR "Pediatrics/urine"[Mesh] ))) OR ((((((children[Title/Abstract]) OR child[Title/Abstract]) OR pediatric[Title/Abstract]) OR paediatrics[Title/Abstract]) OR adolescent[Title/Abstract]) OR toddler[Title/Abstract]))) OR childhood[Title/Abstract]) OR childhood[MeSH Terms])) AND ((((((((("Cystitis"[Mesh]) OR "Pyelonephritis"[Mesh]) OR (( "Bacteriuria/diagnosis"[Mesh] OR "Bacteriuria/epidemiology"[Mesh] OR "Bacteriuria/pathology"[Mesh] OR "Bacteriuria/physiology"[Mesh] OR "Bacteriuria/physiopathology"[Mesh] OR "Bacteriuria/therapy"[Mesh] ))) OR (( "Urinary Tract Infections/diagnosis"[Mesh] OR "Urinary Tract Infections/drug therapy"[Mesh] OR "Urinary Tract Infections/epidemiology"[Mesh] OR "Urinary Tract Infections/etiology"[Mesh] OR "Urinary Tract Infections/microbiology"[Mesh] OR "Urinary Tract Infections/prevention and control"[Mesh] OR "Urinary Tract Infections/urine"[Mesh] ))) OR ((((urinary tract infection[Title/Abstract]) OR lower urinary tract infection[Title/Abstract]) OR cystitis[Title/Abstract]) OR pyelonephritis[Title/Abstract]))) OR ((((( "Vesico-Ureteral Reflux/anatomy and histology"[Mesh] OR "Vesico-Ureteral Reflux/complications"[Mesh] OR "Vesico-Ureteral Reflux/diagnosis"[Mesh] OR "Vesico-Ureteral Reflux/drug therapy"[Mesh] OR "Vesico-Ureteral Reflux/epidemiology"[Mesh] OR "Vesico-Ureteral Reflux/microbiology"[Mesh] OR "Vesico-Ureteral Reflux/physiology"[Mesh] OR "Vesico-Ureteral Reflux/physiopathology"[Mesh] OR "Vesico-Ureteral Reflux/prevention and control"[Mesh] OR "Vesico-Ureteral Reflux/therapy"[Mesh] OR "Vesico-Ureteral Reflux/urine"[Mesh] ))) OR "Vesico-Ureteral Reflux"[Mesh]) OR (((vesicoureteral reflux[Title/Abstract]) OR vesicourethral reflux[Title/Abstract]) OR vesicoureteric reflux[Title/Abstract])))) AND (((("Fecal Incontinence/epidemiology"[Mesh]) OR "Encopresis/epidemiology"[Mesh]) OR (( "Constipation/epidemiology"[Mesh] OR "Constipation/urine"[Mesh] ))) OR (((((((((bowel bladder dysfunction[Title/Abstract]) OR voiding dysfunction[Title/Abstract]) OR dysfunctional voiding[Title/Abstract]) OR dysfunctional elimination syndrome[Title/Abstract]) OR constipation[Title/Abstract]) OR fecal incontinence[Title/Abstract]) OR encopresis[Title/Abstract]) OR lower urinary tract symptoms[Title/Abstract]) OR lower urinary tract dysfunction[Title/Abstract]))) |
| --- | --- |
| Embase | 'child':ti,ab,kw OR 'childhood':ti,ab,kw OR 'pediatrics':ti,ab,kw OR 'paediatric':ti,ab,kw OR 'adolescent':ti,ab,kw OR 'toddler':ti,ab,kw AND ([adolescent]/lim OR [child]/lim OR [infant]/lim OR [preschool]/lim OR [school]/lim OR [young adult]/lim) AND ('Article'/it OR 'Article in Press'/it OR 'Conference Abstract'/it OR 'Conference Paper'/it) AND ('clinical article'/de OR 'clinical trial'/de OR 'cohort analysis'/de OR 'comparative study'/de OR 'controlled clinical trial'/de OR 'controlled study'/de OR 'cross-sectional study'/de OR 'double blind procedure'/de OR 'human'/de OR 'human tissue'/de OR 'intermethod comparison'/de OR 'major clinical study'/de OR 'medical record review'/de OR 'methodology'/de OR 'multicenter study'/de OR 'open study'/de OR 'prospective study'/de OR 'questionnaire'/de OR 'randomized controlled trial'/de OR 'retrospective study'/de OR 'study design'/de)AND ('abdominal pain'/de OR 'bladder dysfunction'/de OR 'congenital malformation'/de OR 'constipation'/de OR 'dysuria'/de OR 'enuresis'/de OR 'feces incontinence'/de OR 'fever'/de OR 'hematuria'/de OR 'hydronephrosis'/de OR 'incontinence'/de OR 'lower urinary tract symptom'/de OR 'micturition disorder'/de OR 'nocturnal enuresis'/de OR 'overactive bladder'/de OR 'postoperative complication'/de OR 'pyelonephritis'/de OR 'recurrent infection'/de OR 'urinary tract infection'/de OR 'urine incontinence'/de OR 'urine retention'/de OR 'vesicoureteral reflux'/de OR 'vomiting'/de) AND [embase]/lim NOT ([embase]/lim AND [medline]/lim) |
| CENTRAL | #1 (edema*):ti,ab,kw OR (oedema):ti,ab,kw OR (swelling):ti,ab,kw (Word variations have been searched)  #2 MeSH descriptor: [Edema] explode all trees  #3 #1 or #2  #4 (nephrotic syndrome):ti,ab,kw OR ("minimal-change disease"):ti,ab,kw OR (minimal change lesion):ti,ab,kw AND ("focal segmental glomerulosclerosis"):ti,ab,kw (Word variations have been searched)  #5 MeSH descriptor: [Nephrotic Syndrome] explode all trees  #6 MeSH descriptor: [Nephrosis, Lipoid] explode all trees  #7 MeSH descriptor: [] explode all trees  #8 #4 or #5 or #6 or #7  #9 (bladder bowel dysfunction):ti,ab,kw OR ("voiding dysfunction"):ti,ab,kw OR (dysfunctional voiding):ti,ab,kw OR (dysfunctional elimination syndrome):ti,ab,kw OR (lower urinary tract dysfunction):ti,ab,kw (Word variations have been searched)  #10 MeSH descriptor: [Lower Urinary Tract Symptoms] explode all trees  #11 (urinary tract infection*):ti,ab,kw OR (lower urinary tract infection):ti,ab,kw OR (pyelonephritis*):ti,ab,kw OR (cystitis):ti,ab,kw (Word variations have been searched)  #12 MeSH descriptor: [Urinary Tract Infections] explode all trees  #13 MeSH descriptor: [Pyelonephritis] explode all trees  #14 MeSH descriptor: [Cystitis] explode all trees  #15 ("vesicoureteral reflux"):ti,ab,kw OR (vesicourethral reflux):ti,ab,kw OR (vesicoureteric reflux):ti,ab,kw OR (vesicourethral reflux):ti,ab,kw OR (VUR):ti,ab,kw (Word variations have been searched)  #16 MeSH descriptor: [Vesico-Ureteral Reflux] explode all trees  #17 (constipation):ti,ab,kw OR ("bowel dysfunction"):ti,ab,kw OR ("fecal incontinence"):ti,ab,kw OR (encopresis):ti,ab,kw (Word variations have been searched)  #18 MeSH descriptor: [Constipation] explode all trees  #19 MeSH descriptor: [Fecal Incontinence] explode all trees  #20 MeSH descriptor: [Encopresis] explode all trees  #21 #9 or #10 or #17 or #18 or #19 or #20  #22 # 11 or #12 or #13 or #14 or #15 or #16  #23 #21 AND #22  #24 (children):ti,ab,kw OR (pediatrics):ti,ab,kw OR ("Child"):ti,ab,kw OR (adolescent):ti,ab,kw (Word variations have been searched)  #25 MeSH descriptor: [Child] explode all trees  #26 #24 or #25  #27 #23 AND #26 |
